# Recent Advances of NIR-II Emissive Semiconducting Polymer Dots for In Vivo Tumor Fluorescence Imaging and Theranostics

**DOI:** 10.3390/bios12121126

**Published:** 2022-12-05

**Authors:** Qidong Wei, Dingshi Xu, Tianyu Li, Xuehan He, Jiasi Wang, Yi Zhao, Lei Chen

**Affiliations:** 1 School of Biomedical Engineering, Sun Yat-Sen University, Shenzhen 518107, China; 2 School of Pharmaceutical Sciences, Sun Yat-Sen University, Shenzhen 518107, China

**Keywords:** semiconducting polymer dots, NIR-II, fluorescence probes, tumor imaging, tumor theranostics

## Abstract

Accurate diagnosis and treatment of tumors, one of the top global health problems, has always been the research focus of scientists and doctors. Near-infrared (NIR) emissive semiconducting polymers dots (Pdots) have demonstrated bright prospects in field of in vivo tumor fluorescence imaging owing to some of their intrinsic advantages, including good water-dispersibility, facile surface-functionalization, easily tunable optical properties, and good biocompatibility. During recent years, much effort has been devoted to developing Pdots with emission bands located in the second near-infrared (NIR-II, 1000–1700 nm) region, which hold great advantages of higher spatial resolution, better signal-to-background ratios (SBR), and deeper tissue penetration for solid-tumor imaging in comparison with the visible region (400–680 nm) and the first near-infrared (NIR-I, 680–900 nm) window, by virtue of the reduced tissue autofluorescence, minimal photon scattering, and low photon absorption. In this review, we mainly summarize the latest advances of NIR-II emissive semiconducting Pdots for in vivo tumor fluorescence imaging, including molecular engineering to improve the fluorescence quantum yields and surface functionalization to elevate the tumor-targeting capability. We also present several NIR-II theranostic Pdots used for integrated tumor fluorescence diagnosis and photothermal/photodynamic therapy. Finally, we give our perspectives on future developments in this field.

## 1. Introduction

**Figure 1:**
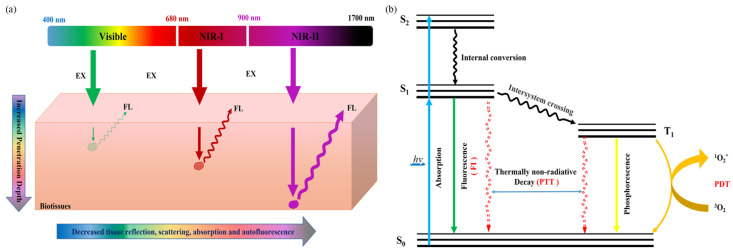
(**a**) Schematic illustration of the fluorescence signals with different wavelengths in biological tissues and (**b**) the mechanism of the fluorescence-generation process and photothermal/photodynamic therapy.

In vivo biomedical imaging is a technique that generates internal images of the body by non-invasive means and is commonly used in clinical analysis and medical interventions. Various biological imaging modalities have been validated through previous research and clinical practice, such as photoacoustic imaging (PAI) [1,2,3], fluorescence imaging (FI) [4,5], computed tomography (CT) [6], positron emission tomography (PET) [7,8], and single-photon emission computed tomography (SPECT) [9]. In comparison to these clinical imaging techniques, fluorescence imaging (FI) has the advantages of real-time imaging, high sensitivity, rapid feedback, good spatial and temporal resolution, and good biocompatibility [10,11]. Despite the many advantages of FI imaging, in vivo FI is not optimal in the visible (400–680 nm) and the first near-infrared (NIR-I, 680–900 nm) regions due to photon attenuation caused by biological tissues and autofluorescence [12,13]. In recent years, as shown in Figure 1a, the second near-infrared (NIR-II, 1000–1700 nm) region has attracted much attention for its advantages of greatly improved penetration depth (5–20 mm), spatial and temporal resolution (25 mm and 20 ms), high signal-to-background ratio (SBR), low tissue absorption, and minimal autofluorescence interference [14,15]. When NIR-II light passes through tissues, the interaction between photons and tissues is significantly reduced, and the photons can penetrate into deeper tissues, so as to achieve better surgical guided anatomy (Figure 1b). For this purpose, various NIR-II emissive fluorescent probes, such as metal nanoparticles [16,17,18], inorganic semiconductor nanoparticles [19,20], small molecular-based nanoparticles [20,21], and aggregation-induced emission (AIE) nanoparticles [22,23], have been established.

**Figure 2:**
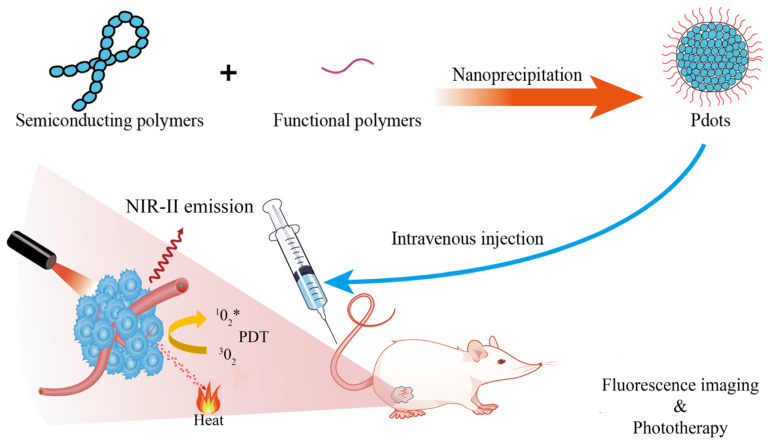
Illustration the nanoformulation of Pdots and their applications for tumor fluorescence imaging and phototherapy.

Very recently, semiconducting polymer dots (Pdots) have attracted extensive attention for in vivo fluorescence imaging applications [24]. Pdots are types of organic polymer-based nanoparticles, mainly composed of π-conjugated hydrophobic polymer as light absorber and fluorescent emitter [25], with additional non-conjugated polyethylene glycol (PEG)-based amphiphilic polymers as a surfactant to stabilize the nanoparticles in aqueous solution and to avoid non-specific binding of Pdots to biological species [26]. Pdots can be prepared by nanoprecipitation, mini-emulsion polymerization, self-assembly of amphiphilic block copolymers, or a microfluidic approach, which are detailed in previous reviews [24,27,28]. Usually, the mass or volume fraction of the conjugated polymer in a single Pdot must be greater than 50% and with a diameter less than 40 nm [29]. Compared with nanoparticles prepared by traditional materials such as single carbon nanotubes (SWCNTs) [30], lanthanide metals [31], and small organic molecules [14,32], Pdots have demonstrated significant fluorescence properties in biological studies, such as good biocompatibility [33,34] and photostability [35,36], fast radiation decay rate [37,38], large absorption coefficient [39], ultrahigh single-particle brightness [40]. Combined with the adjustable structures and easy surface functionalization properties, Pdots have become a promising diagnostic and therapeutic platform for biosensors [41,42], cell labeling [43,44], tissue imaging [45,46], phototherapy applications (Figure 2) [47], and the nanocarriers for drug delivery, which have been well-summarized by recent reviews [48,49,50,51]. Despite the above-mentioned advantages and the great progress achieved, Pdots still have some problems to be solved for better biologic applications. For example, it is difficult to prepare Pdots with uniform nano-size by a simple nanoprecipitation method. Second, it is difficult to obtain small enough Pdots (≤10 nm, with a high content of conjugated polymer) to enhance their tumor targeting/permeability ability, and little work has been reported on their metabolism and long-term biosafety in vivo. Thirdly, the fluorescence quantum efficiency (*Φ*_f_) of the Pdot probe is usually lower than comparable small-molecular probes or inorganic metallic quantum dots (Qdots), especially for NIR-II emissive Pdots.

Many NIR-II emissive Pdots have been presented for deep-tissue FI with high spatial temporal resolution, such as blood vessels, bones, lymph node, solid tumors, through-skull imaging, and cell tracking, which are referenced in recent reviews [52,53]. Herein, we will focus on the latest reported NIR-II Pdots for in vivo tumor imaging, and in particular on the molecular engineering to optimize the fluorescence quantum yields and surface functionalization to promote the ability of active tumor targeting. Some of the NIR-II theranostic Pdots used for integrated FI diagnosis and phototherapy are also included. The properties and their applications of the NIR-II Pdots summarized in this review are listed in Table 1. Finally, we will discuss the future directions and challenges in this field.

## 2. Molecular Engineering of Efficient NIR-II Pdots for In Vivo Tumor FI

As mentioned above, light with wavelengths in the NIR-II region has a high development potential for in vivo imaging of tumors due to its small absorption and scattering in animal tissues, better tissue penetration and higher spatial resolution. However, NIR-II emissive Pdots usually exhibit low *Φ*_f_ due to an intrinsic small energy bandgap between the lowest unoccupied molecular orbital (LUMO) and the highest occupied molecular orbital (HOMO) of conjugated polymers, as well as the severe aggregation-caused quenching (ACQ) effect in the Pdot state, both of which facilitate non-radiative decay, resulting in low *Φ*_f_ of NIR-II Pdots [49,62,63]. Therefore, one important challenge is to design NIR-II Pdots with high *Φ*_f_, to realize high brightness in vivo and hence better SBR of fluorescence imaging.

**Figure 3:**
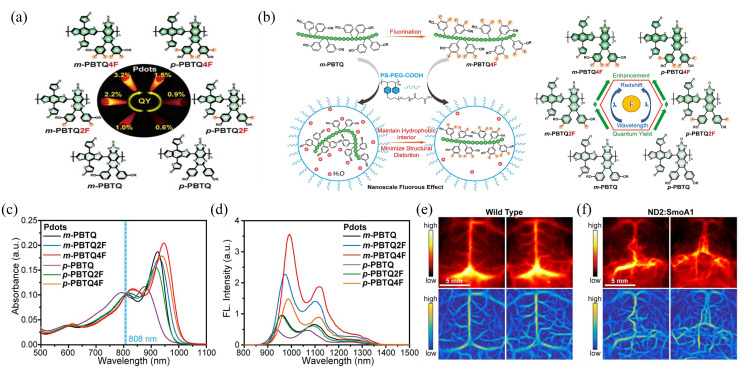
(**a**) Fluorescence quantum yield of Pdots prepared by different fluorination strategies. (**b**) Schematic representation of the nanoscale fluorine effect. (**c**) Absorption spectra of various Pdots. (**d**) Fluorescence spectra of various Pdots. (**e**) In vivo NIR-II fluorescence images (top panel) and Hessian-matrix-enhanced images (bottom panel) of cerebral vasculature of wild-type C57BL/6 mice. (**f**) In vivo NIR-II fluorescence images (top panel) and Hessian-matrix-enhanced images (bottom panel) of cerebral vasculature of ND2:SmoA1 mice. Reproduced with permission from ref. [54]. Copyright 2020 Wiley-VCH GmbH.

To develop NIR-II Pdot probes with high *Φ*_f_, Liu et al., proposed a fluorination strategy to design semiconducting polymers to optimize the *Φ*_f_ of corresponding Pdot probes [54]. As shown in Figure 3a, using benzodithiophene (BDT) as electron donor (D) and triazole [4,5-g]-quinoxaline (TQ) derivatives as electron acceptor (A), the team synthesized two sets of fluorine-substituted D-A type semiconducting polymers based on the two fluorine substitution modes, which are named as m-PBTQ, m-PBTQ2F, m-PBTQ4F, m-PBTQ4F, with alkoxy chains anchored at the meta site of benzene and p-PBTQ, p-PBTQ2F, and p-PBTQ4F, with alkoxy chains anchored to the para position of benzene. The semiconducting polymers and amphiphilic polystyrene polymer (PS-PEG-COOH) were prepared into Pdots by the classic nano-precipitation method. The *Φ*_f_ of obtained m-series Pdots (1.0%, 2.2%, and 3.2%) were consistently higher than those of p-series Pdots (0.6%, 0.9%, and 1.5%), mainly because the different alkoxy positions on TQ affected the hydrophobicity and steric hindrance of molecules. Additionally, the *Φ*_f_ of Pdots was increased with more fluorine atoms on the TQ acceptor. As a result, the tetrafluorinated m-PBTQ4F Pdots yielded the highest *Φ*_f_ of 3.2%, which was three times higher than that of the non-fluoridated counterpart and six times higher than that of IR26. Liu et al. attributed the fluorescence enhancement in the fluorinated Pdots to the nanoscale fluorous effect that increases the planarity of the conjugated backbone and minimizes the structure distortion between the excited-state and ground-state, thus decreasing the nonradiative decay rates (Figure 3b). The fluorescence intensity of m-PBTQ4F Pdots remained at 80% of the initial state under 120 min laser irradiation, indicating good photostability. The authors suggest that fluorination of PBTQ polymers can effectively modulate the optical properties of the resulting Pdot in several ways, including energy-level reduction and fluorescence enhancement. Quantitative cranial and scalp-puncture imaging of brain tumor vasculature in vivo demonstrated that m-PBTQ4F Pdot images showed better imaging performance than non-fluoridated m-PBTQ images, and could distinguish normal, uniform, and orderly brain blood vessels from the images, and also clearly differentiate uneven and chaotic distribution. Brain-tumor vasculature with serpentine course and irregular branching are shown in Figure 3e,f. These results indicate that fluorinated Pdots have good photostability and high brightness, and have great development potential in the diagnosis and detection of brain tumors.

**Figure 4:**
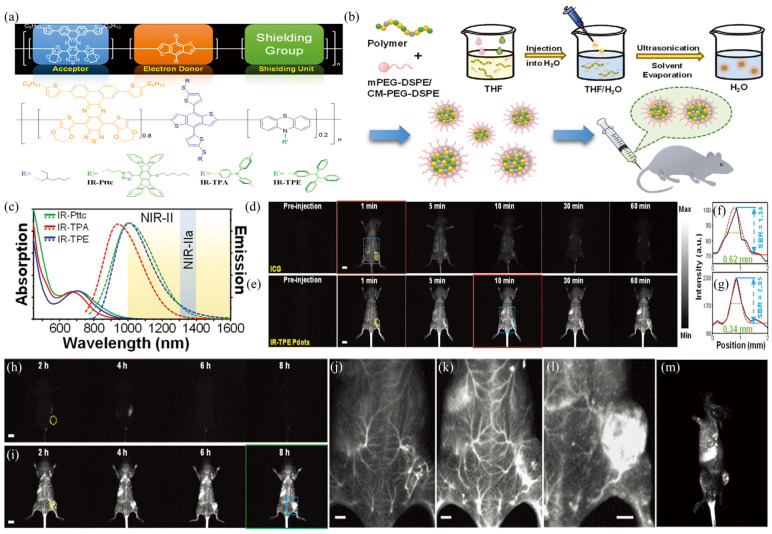
(**a**) Molecular design of three semiconducting polymers. (**b**) Schematic diagram of preparation of the three Pdots. (**c**) Absorption and emission spectra of IR-PTTC, IR-TPA and IR-TPE Pdots in aqueous solution. (**d**,**e**) Fluorescence imaging of mice in vivo within one hour after ICG (**d**) and IR-TPE Pdots (**e**) injection. The yellow circle indicates the location of the tumor. (**f**) Cross-sectional intensity profile along the red line in (**d**). (**g**) Cross-sectional intensity profile along the red line in (**e**). (**h**–**j**) Fluorescence imaging of mice in vivo within 2–8 h after ICG (**h**) and IR-TPE Pdots (**i**) injection. (**j**) Enlarged view of the area in the blue square in (**d**). (**k**) Enlarged view of the area in the blue square in (**e**). (**l**) Enlarged view of the area in the blue square in (**i**). (**m**) Reconstructed 3D mapping of whole-body mouse at 6 h post-injection of IR-TPE Pdots. Reproduced with permission from ref. [55]. Copyright 2021 Wiley-VCH GmbH.

To solve the low *Φ*_f_ problem of NIR-II probes arising from the ACQ effect, Li et al., proposed two strategies to develop efficient NIR-II Pdots, by incorporating an anti-ACQ unit or an aggregation-induced emission (AIE) segment into the polymer backbone [55]. The authors first designed a conjugated polymer skeleton using [1,2,5]thiadiazolo [3,4-g]quinoxaline (TQ) as a strong A unit and alkylthio-thiophene-substituted benzodithiophene as the D unit. A phenothiazines with Pttc (anti-ACQ), TPA (AIE), or TPE (AIE) unit was then added to the semiconducting polymer backbone (Figure 4a). The amphiphilic lipids were subsequently made into Pdots by a nanoprecipitate with semiconducting polymers (Figure 4b). The results showed that IR-PTTC Pdots had the lowest *Φ*_f_ of 4.9% among the three Pdots, while IR-TPA and IR-TPE Pdots had *Φ*_f_ of 6.7% and 14%, respectively. To the best of our knowledge, the IR-TPE Pdot is, to date, the most efficient NIR-II emissive Pdot. At the same time, IT-TPE has a strong absorption at 700 nm, and the maximum emission peak is 1010 nm (Figure 4c). Due to the high *Φ*_f_ of IR-TPE Pdots, the authors used these Pdots for subsequent bioimaging. First, the authors functionalized the surface of IR-TPE Pdots with folic acid so that specific cancer cells with folate receptors could internalize them. Subsequently, Pdots were injected intravenously into mice through the tail vein, and the SBR reached 2.42 when equipped with a 1400 nm long-pass filter. At the same time, their fluorescence intensity remained more than 80% of their original intensity after 20 min of continuous UV irradiation. These results indicate that Pdots are very light resistant and suitable for long-term fluorescence imaging or tracking. The authors performed in vivo tumor imaging using IR-TPE Pdots and ICG in live mice bearing 4T1 tumors and compared the performance of these two probes (Figure 4d,e). At the same time, 3D tumor mapping was performed in vivo in tumor-bearing mice 6 h after injection (Figure 4h,l). The images in the film were reconstructed from a series of images with different rotation angles from −45° to 45°, from which we could easily identify the location of the tumor (Figure 4m).

## 3. Active-Tumor-Targeting NIR-II Pdots for In Vivo Tumor FI

**Figure 5:**
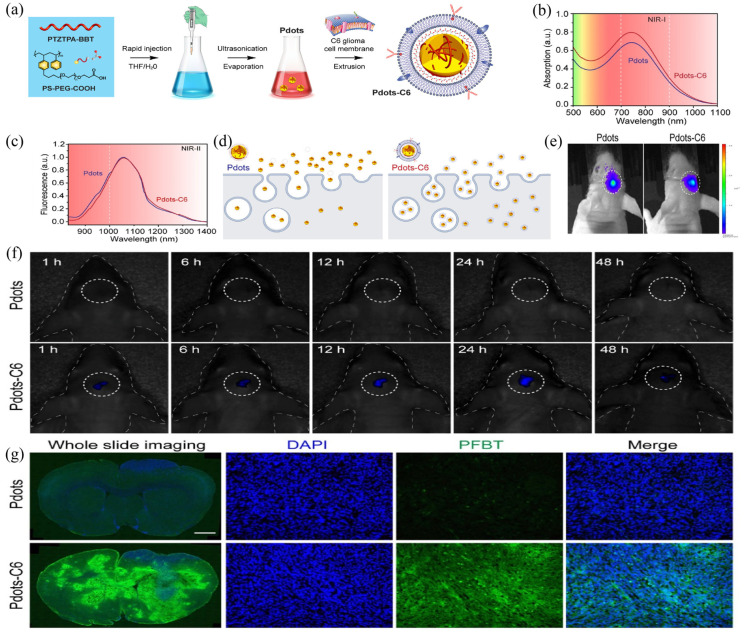
(**a**) Preparation process of Pdots-C6. (**b**) Absorption spectra of Pdots and Pdots-C6. (**c**) Fluorescence spectra of Pdots and Pdots-C6. (**d**) Schematic diagram of cell uptake difference between Pdots and Pdots-C6. (**e**) In vivo fluorescence images of glioma-bearing mice. (**f**) In vivo NIR-II fluorescence imaging of glioma with Pdots and Pdots-C6 administration at different time points post-injection. (**g**) Brain tissue section 24 h post-injection. Green: PFBT; blue: cell nuclei stained with DAPI. Reproduced with permission from ref. [56]. Copyright 2021 Elsevier.

According to previous work, Pdot probes can not only accumulate in tumor sites through an enhanced permeability and retention (EPR) effect, but can also gather at normal organs such as the liver, spleen, or lymph nodes [64,65]. To further improve the tumor-targeting capability of Pdots, Men et al. recently constructed a novel bionic Pdot—Pdots-C6—using a natural cell membrane as a shell for nanoparticles [56]. The authors first developed a semiconducting polymer PTZTPA-BBT using triphenylamine (TPA) functionalized phenothiazine (PTZ) as a D unit and benzo [1,2-c:4,5-c’]bis [1,2,5]thiadiazole (BBT) as a strong A unit. Then PTZTPA-BBT underwent nanoprecipitation with PS-PEG-COOH to prepare Pdots (Figure 5a). Subsequently, Pdots and purified C6 cell membrane were co-extruded to obtain the cell-membrane-encapsulated Pdots: Pdots-C6. At 808 nm excitation, Pdots-C6 has a maximum emission peak at 1055 nm (Figure 5b,c). The native structure of C6 cell membrane and the antigen on the membrane surface render Pdots-C6 capable of special functions such as antibody targeting recognition, long blood circulation, and immune escape. Membrane proteins on the membrane surface of homologous C6 cells can signal to macrophages to avoid being cleared by macrophages. At the same time, C6 cells had a significantly higher uptake tendency of C6CM-coated Pdots (Figure 5d), which demonstrated the targeting ability of the membrane-coating strategy. After the establishment of the hippocampal orthotopic glioma mouse model, the authors injected Pdots and Pdots-C6 through the tail vein and monitored the fluorescence signal at the tumor site (Figure 5e). The results showed that the accumulation of Pdots-C6 was more pronounced in the tumor region, which was due to the better glioma-targeting ability and blood−brain barrier penetration ability of the C6 cell membrane (Figure 5f,g).

**Figure 6:**
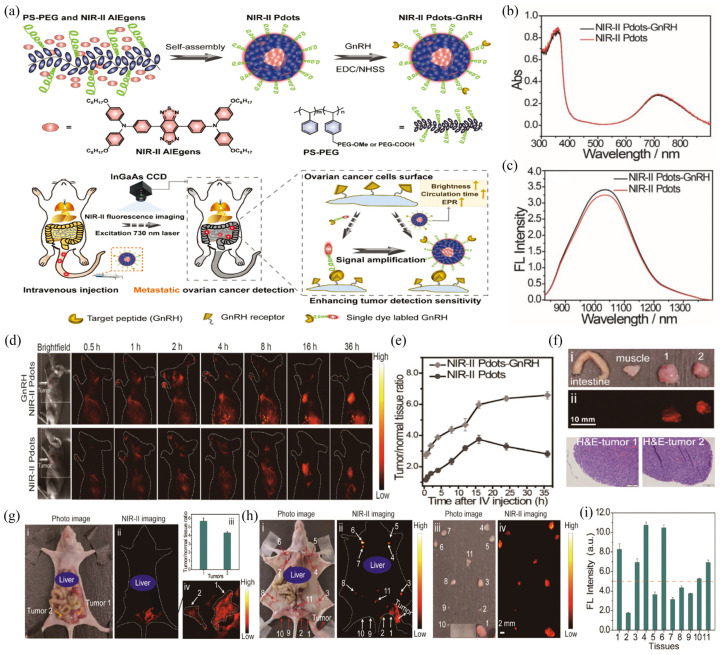
(**a**) Schematic illustration of NIR-II Pdots-GnRH preparation and its use for the detection of metastatic ovarian cancer in vivo. (**b**) Absorption and (**c**) fluorescence spectra of NIR-II Pdots and NIR-II Pdots-GnRH. (**d**) NIR-II fluorescence images of live tumor-bearing mice injected with NIR-II Pdots and NIR-II Pdots-GnRH. (**e**) Tumor/normal tissue fluorescence ratio within 36 h. (**f**) Optical (i) and fluorescent images (ii) of surgically resected tumor tissues and normal tissues (muscle and intestine) guided by NIR-II fluorescence imaging and H&E maps of tumor tissue. (**g**) Optical photographs of peritoneal metastasis model of ovarian adenocarcinoma 48 h after injection and (i) corresponding NIR-II fluorescence bioimaging results (ii), enlarged images of large peritoneal metastases labeled with NIR-II nanoprobes (Numbers 1 and 2), (iii), and mean signal-to-noise ratio of peritoneal metastases (iv). (**h**) (i) Optical photos and (ii) NIR‐II fluorescence images of ovarian lymphatic metastases mode obtained at 48 h post-injection; (iii) optical photos and (iv) NIR‐II fluorescence images of the resected lymph node tissues (No. 1-11) in vitro. (**i**) Mean fluorescence intensity of resected lymph node tissue shown in (**h**). Reproduced with permission from ref. [57]. Copyright 2020 Wiley-VCH GmbH.

Li et al., designed NIR-II Pdots based on NIR-II emitting AIEgens triphenylamine-benzo [1,2-c:4,5-c’]bis([1,2,5]thiadiazole) (BBTD) and amphiphilic polymer PS-PEG to further obtain NIR-II Pdots-GnRH after modification with ovarian-cancer-targeting peptide GnRH for ovarian cancer metastasis detection in vivo (Figure 6a) [57]. NIR-II Pdots-GnRH showed a maximum absorption peak near 710 nm and a maximum emission peak of 1020 nm (Figure 6b,c), with an IR-26 reference and a high *Φ*_f_ of 5.5% in aqueous solution. In in vivo imaging experiments using NIR-II Pdots and NIR-II Pdot-GnRH in a mouse subcutaneous human ovarian adenocarcinoma model, NIR-II Pdots-GnRH exhibited better affinity for tumor tissue (Figure 6d). NIR-II Pdots-GnRH accumulated more rapidly and retained longer in tumor tissues than bare NIR-II Pdots (Figure 6e). Real-time imaging of peritoneal and lymphatic metastasis of ovarian cancer was achieved after tail-vein injection of NIR-II Pdots-GnRH in peritoneal metastasis tumor-bearing mice and lymphatic metastasis tumor-bearing mice. The results showed that the tumor boundary of NIR-II Pdots-GnRH-treated peritoneal metastatic tumor-bearing mice was clearly visible, and the SBR value of the tumor area was as high as 5.5. However, after NIR-II Pdots-GnRH treatment, the whole lymph nodes of mice showed NIR-II fluorescence signal (Figure 6i), and the lymph node metastases showed a stronger mean fluorescence intensity than normal lymph nodes (Figure 6h).

## 4. NIR-II Pdots as Tumor Theranostic Platforms

Pdots can not only achieve real-time diagnostics owing to their excellent optical properties, but can also serve for phototherapeutic techniques [66,67], and even for stimuli-responsive drug delivery. Hence, Pdots show great potential as theranostic platforms. Herein, we will introduce the advances of theranostic (FI + PTT/PDT) platforms based on NIR-II Pdots.

**Figure 7:**
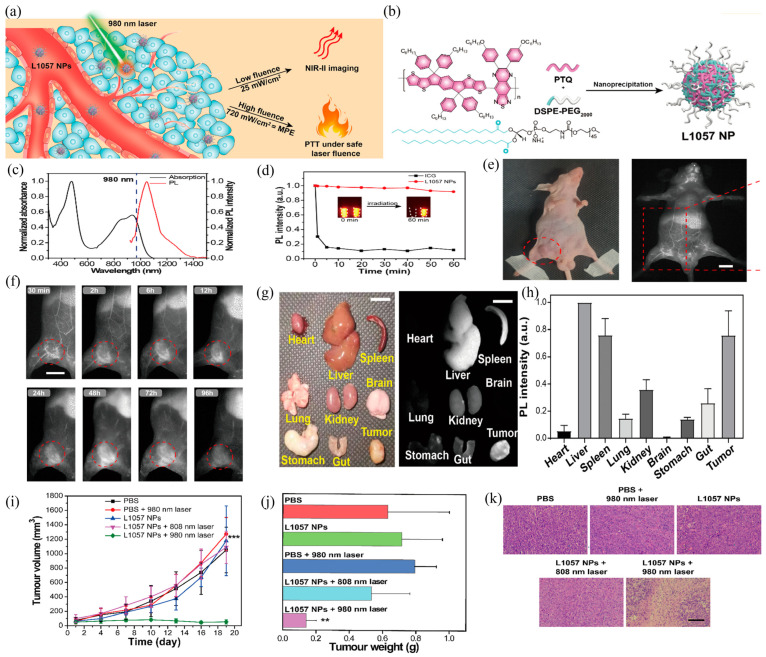
(**a**) Schematic representation of L1057 Pdots as therapeutic diagnostic agents. (**b**) Chemical structures of PTQ and DSPE-PEG2000 and mode of preparation as L1057 Pdots. (**c**) Normalized fluorescence and absorption spectra of L1057 Pdots in Milli-Q water. (**d**) Photostability measurements of L1057 Pdots and ICG in water under 808 nm laser irradiation. (**e**) Breast-tumor-bearing nude mice. (**f**) NIR-II fluorescence imaging of L1057 Pdots at various time points over 96 h in breast-tumor-bearing mice excited with 980 nm (25 mW cm^−2^, 20 ms). (**g**) Bright-field and NIR-II images of individual organs. (**h**) Normalized standard fluorescence intensity of each organ (liver fluorescence intensity as the standard, n = 3). (**i**) Tumor volume and (**j**) tumor weight at various time points after different treatment regimens. (**k**) H&E staining of tumor tissue after 18 days of PTT treatment. (Results are presented as the mean ± S.D., n = 5). Statistical significance was calculated using one-way ANOVA with the Tukey posthoc test. ** *p* < 0.01, *** *p* < 0.001. Reproduced with permission from ref. [58]. Copyright 2020 American Chemical Society.

Yang’s group reported a L1057 Pdots system based on PTQ semiconducting polymer and DSPE-PEG2,000 surfactant (Figure 7a,b), whose emission spectrum almost completely lies in the NIR-II window with a peak of 1057 nm (Figure 7c), and has a high mass extinction coefficient (ε = 18 L g^−1^ cm^−1^) and a *Φ*_f_ of 1.25% [58]. Compared to the small-molecular ICG fluorescent probe, L1057 Pdot shows pretty good photostability, which has almost no photobleaching during 60 minutes’ excitation (Figure 7d). PTQ polymer adopts a role as a weak electron acceptor and its energy band gap is narrower than that of NIR-II conjugated polymer reported previously. As a result, the absorption band of L1057 Pdots was red shifted with a peak around at 980 nm, which means higher maximum permissible exposure (MPE) can be obtained. After verification, the commonly used 980 nm and 808 nm excitation laser sources are both suitable for biological imaging. The accurate in vivo tumor imaging ability of L1057 Pdots was evaluated in the 4T1 tumor model (Figure 7e). Through the representative FI of different organs shown in Figure 7h, it can be found that the Pdots were mainly accumulated in the liver, spleen, tumor, and kidney. The tumor fluorescence started to decrease at 48 h (Figure 7f), indicating that L1057 Pdots could be effectively cleared by the circulation system, and L1057 Pdots were also applied to PTT treatment due to their excellent photo-thermal power-conversion capability. As shown in Figure 7i,k, after five different treatments, the L1057 Pdots + 980 nm laser irradiation showed the most significant tumor treatment effect, with no recurrence within 18 days of observation. The H&E staining also proved that the L1057 Pdots + 980 nm laser irradiation group had the best killing effect on tumor cells, and the cells in the other groups were in good condition.

**Figure 8:**
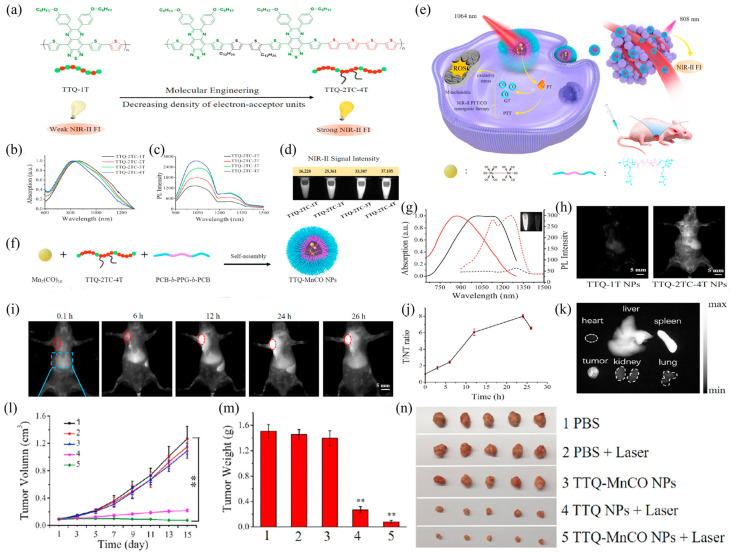
(**a**) Schematic representation of the mechanism by which conjugated polymer electron acceptor density is modulated to enhance NIR-II fluorescence of TTQ-2TC-1T, TTQ-2TC-2T, TTQ-2TC-3T, and TTQ-2TC-4T at the same concentration in toluene. (**b**) Absorption/1064 nm excitation/0.5 h/26 h/1064 nm. (**c**) Fluorescence spectra. (**d**) NIR-II fluorescence signal. (**e**) NIR-II fluorescence guided treatment of MCF-7 tumors under 1064 nm excitation. (**f**) Schematic diagram of TTQ-MnCO Pdots preparation. (**g**) Absorption spectra (TTQ-1T Pdots, black line; TTQ-2TC-4T Pdots, red line) and fluorescence spectra (TTQ-1T Pdots, black dotted line; TTQ-2TC-4T Pdots, red dotted line). (**h**) NIR-II fluorescence images after 0.5 h injection of TTQ-1T Pdots and TTQ-2TC-4T Pdots. (**i**) NIR-II fluorescence pattern of MCF-7 tumor mice at different times after TTQ-MnCO Pdots injection. (**j**) Fluorescence signal intensity ratio compared with normal tissue in (**i**). (**k**) Fluorescence images of isolated organs after 26 h. (**l**) Tumor-volume curves and (**m**) weight changes of different groups of MCF-7 tumor-bearing mice exposed to 1064 nm laser and (**n**) tumor photos taken after 15 days. Statistical significance was calculated using one-way ANOVA with the Tukey posthoc test. ** *p* < 0.01. Reproduced with permission from ref. [59]. Copyright 2022 Elsevier.

Fan et al., proposed a strategy to enhance the brightness of NIR-II Pdot by introducing more electron-donating units, which means less electron-withdrawing units in a D–A type conjugated polymer [59]. Adopting this idea, they synthesized a series of conjugated polymers with an increased number of thiophene units in the main chain to strengthen the radiative decay (Figure 8a). These were named TTQ-2TC-1T, TTQ-2TC-2T, TTQ-2TC-3T, and TTQ-2TC-4T. A long alkyl substituted dithiophene segment was introduced to improve the solubility in organic solvents (Figure 8b). The corresponding TTQ-2TC-4T Pdots exhibit the best NIR II fluorescence in aqueous solution (Figure 8e). TTQ-2TC-4T has a typical NIR-II emission peak at 1038 nm (Figure 8b,c). After tail-vein injection into tumor-bearing mice, TTQ-2TC-4T Pdots show much brighter fluorescence than that of TTQ-1T Pdots (Figure 8h). Moreover, theranostic TTQ-MnCO Pdots can be obtained by co-loading with a thermal-responsive Mn_2_(CO)_10_ compound on an amphiphilic polymer (PCB-b-PPG-b-PCB) (Figure 8f,g). In MCF-7 tumor-bearing mice injected with TTQ-MnCO Pdots via the tail vein, an obvious fluorescence signal was observed at the tumor site 6 h post-injection, reaching maximum brightness at 24 h. The TTQ-MnCO Pdots were then metabolized through the hepatobiliary system (Figure 8i). TTQ-MnCO Pdots are guided by NIR-II fluorescence for PTT/CO synergistic treatment, which provides a phototherapeutic strategy with great potential for cancer treatment. What is more, this might be a generalized way to reduce the electron-withdrawing groups in the conjugated polymer to optimize the brightness of NIR-II Pdots. As shown in Figure 8l,m, TTQ-MnCO Pdots injected into the tail vein under laser irradiation had obvious tumor killing effect. It is worth noting that the TTQ Pdots group also had a tumor-killing effect under laser irradiation, due to its PTT effect. The different therapeutic effects of TTQ Pdots and TTQ-MNCO Pdots under laser irradiation also further demonstrate PTT/CO synergistic treatment.

**Figure 9:**
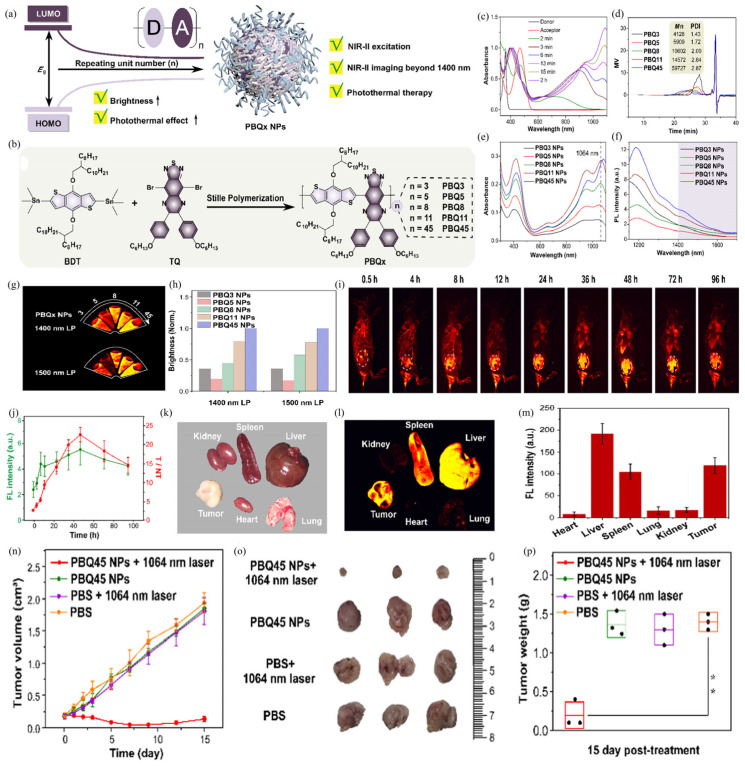
(**a**) Schematic representation of the effect of repetitive units on semiconducting polymers. (**b**) Schematic representation of semiconducting polymer synthesis with different repeat units. (**c**) UV-Vis-NIR spectra of mixtures dissolved in chloroform at different time points. (**d**) GPC test results of polymers. The same concentration of PBQx Pdots (**e**) and V-Vis-NIR. (**f**) Emission spectrum with an excitation wavelength of 1064 nm. (**g**) Fluorescence image and (**h**) quantitative fluorescence analysis. (**i**) Fluorescence images of 4T1 tumor-bearing mice after injection of PBQ45 Pdots. (**j**) Fluorescence intensity and T/NT ratio of the tumor region at each time point. (**k**) Bright-field image. (**l**) Fluorescence image and (**m**) fluorescence intensity of isolated organs after 96 h. (**n**) Tumor volume curves of tumor-bearing mice after treatment with different groups of PTT. (**o**) Photographs of tumors excised from mice and (**p**) tumor weights at the end of treatment. Statistical significance was calculated using one-way ANOVA with the Tukey posthoc test. ** *p* < 0.01. Reproduced with permission from ref. [60]. Copyright 2022 American Chemical Society.

The fluorescence and photothermal therapy effect of Pdots can be simultaneously improved by adjusting the degree of polymerization (DP) of the semiconducting polymers. Huang et.al reported a semiconducting polymer-based therapeutic nano-agent (PBQx Pdots) and demonstrated its potential application in fluorescence imaging of the NIR-II region above 1400 nm as well as in tumor therapy (Figure 9a) [60]. Based on the D–A design strategy, they increased the effective conjugate length of the polymer structure by increasing the repeat units of the polymer backbone, and thus affected the fluorescence brightness and photothermal properties (Figure 9b). The reaction time and UV-Vis-NIR absorption spectra were controlled to monitor the reaction process and synthesize PBQx with different DPs (Figure 9c). According to the results of gel-permeation chromatography (GPC), the number of repeat units of each polymer was calculated (Figure 9d), and PBQx Pdots were prepared by the nano-precipitation method. The results showed that PBQ45 Pdots with the highest DP possess a much more red-shifted absorption band and a larger mass extinction coefficient (ε), and also display much better brightness and photothermal effects than PBQ5 Pdots and PBQ3 Pdots (Figure 9e,f). Under the guidance of NIR-II fluorescence at wavelengths beyond 1400 nm, PBQ45 can accurately distinguish solid tumors in vivo, demonstrating that PBQ45 is a long-wavelength-emissive nanoprobe with the potential for tumor imaging (Figure 9g,h). Significant accumulation of PBQ45 Pdots was observed at tumor sites 48 h after intravenous injection into 4T1-bearing mice, and fluorescence intensity was compared in isolated organs 96 h later (Figure 9i,m). In addition, PBQ45 Pdots have good anti-tumor ability when employed as a therapeutic diagnostic agent for NIR-II fluorescence-image-guided PTT (Figure 9i). The photo-thermal conversion of the polymer as well as the extinction coefficient tend to be consistent with the increase of the DPs. As shown in Figure 9n, the volume of the solid tumor in vivo almost did not grow under PBQ45 NPs + 1064 nm laser irritation conditions. While the tumor volume greatly increased when there was only PBS buffer, PBQ45 NPs, or PBS buffer + 1064 nm laser irritation. These results prove the therapeutic effect of PBQ45 NPs by PTT treatment. Generally, this study provides a simple and effective way for simultaneously improving the brightness and photothermal efficiency of theranostic Pdots.

**Figure 10:**
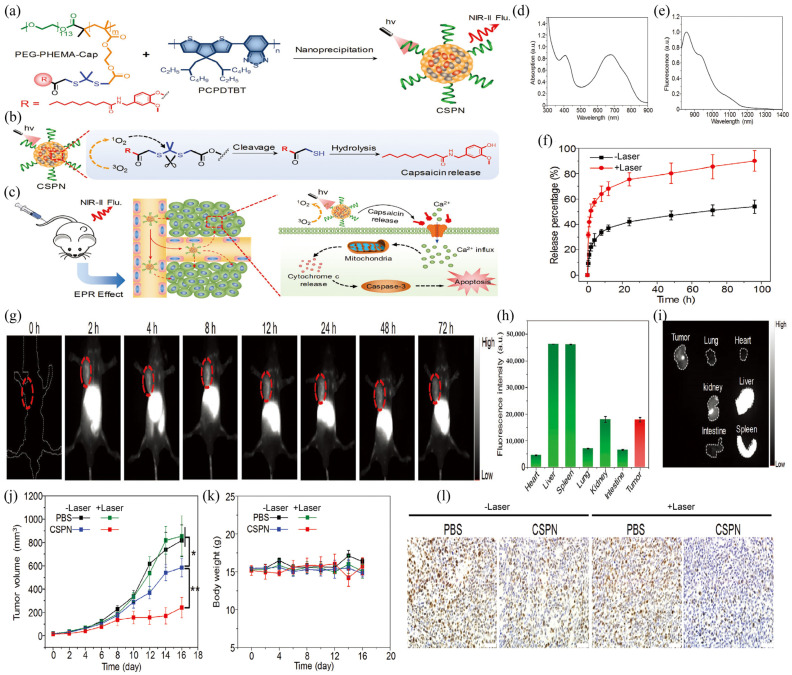
(**a**) Flow chart of CSPN preparation. (**b**) Schematic illustration of capsaicin release under near-infrared light. (**c**) In vivo imaging and schematic diagram of tumor therapy of CSPN. (**d**) Absorption and (**e**) fluorescence spectra of CSPN in PBS. (**f**) Release curve of capsaicin with or without laser irradiation at 635 nm. (**g**) Fluorescent image of NIR-II in tumor-bearing mice (CSPN, 200 μg mL^−1^, 200 μL). (**h**) Fluorescence intensity and (**i**) main tissue photographs from CSPN-injected mice at 72 h. (**j**) Tumor volume and (**k**) body weight changes under different treatments. Statistical significance was calculated using one-way ANOVA with the Tukey posthoc test. * *p* < 0.05, ** *p* < 0.01. (**l**) PCNA staining of tumor tissues collected from tumor-bearing mice under different treatments. Reproduced with permission from ref. [61]. Copyright Wiley-VCH Verlag GmbH.

Photodynamic therapy (PDT) is a new low-invasive, high-efficiency, and non-drug-resistant tumor therapy [68]. Photosensitizer is used to stimulate the body with light of a specific wavelength, and the light energy emitted by the body is absorbed by the oxygen in the surrounding environment, thus producing highly oxidizing reactive oxygen species (ROS). These ROS can effectively induce apoptosis of cancer cells. Previous studies have shown that PDT therapy mediated by NIR-II FL can precisely locate the tumor, thus improving the efficiency of PDT and its anti-tumor effect [69,70]. Fan et al., designed and manufactured a capsaicin-modified semiconductor polymer nanoparticle (CSPN) to regulate the calcium ion channels of cancer cells (Figure 10a,b) [61], enabling calcium overload cancer treatment without the addition of additional calcium (Figure 10c). The nanoparticles were composed of a semiconductor polymer PCPDTBT and a capsaicin-coupled amphiphilic polymer PEG-PHEMA-Cap, which produced a strong fluorescence signal of NIR-II. PCPDTBT can generate singlet oxygen under NIR light excitation, which can not only induce photodynamic therapy (PDT), but also promote the release of capsaicin (Figure 10f). The released capsaicin activates the TRPV1 calcium channel, which is over-expressed in cancer cells, allowing calcium ions outside the cell to flow into the cell. In vitro cell experiments have demonstrated that CSPN can produce singlet oxygen under laser irradiation and promote the release of capsaicin, thus activating TRPV1 to induce calcium overload therapy in cells. Subsequent experiments on tumor-bearing mice showed that the tumor inhibition rate was the highest in mice injected with CSPN and irradiated by laser, reaching 70.7% (Figure 10j). Secondly, considering the slow release of capsaicin in the absence of laser irradiation, a certain tumor inhibition effect was achieved. The mice injected with CSPN without laser irradiation proved this conjecture, with a tumor inhibition rate of 28.7% (Figure 10l). The presentation of this study fully demonstrates the potential of NIR-II semiconducting Pdots as the FI + PDT theranostic nanoplatforms.

## 5. Summary

In general, we have summarized a series of semiconducting Pdots with NIR-II window emission and their applications in tumor imaging and theranostics fields. As a new type of fluorescent probe, NIR-II Pdots have unique advantages to achieve high-precision tumor imaging and cancer treatment with high MPE and penetration depth.

Despite great progress, more research and exploration are needed to adjust and optimize the properties of Pdots. In our view, future work should include the following aspects: (1) optimization of the nanoformulation methods for large-scale, green, and low-cost preparation of Pdots; (2) further improvement of strategies for encapsulation, functionalization, and bioconjugation; (3) designing of novel structures with simultaneous NIR-II absorption and emission bands to promote light penetration depth in vivo, to elevate the light energy utilization efficiency of corresponding applications (*Φ*_f_ for FI, PCE for PTT, and singlet oxygen generation efficiency for PDT); consideration of the mass extinction coefficient of Pdots, which is related to the light absorbing ability; (4) development of small-size Pdots (≤10 nm) and enhancement of their tumor-targeting and penetration ability; (5) development of multimodal imaging and construction of theranostic systems and integrated therapeutic platforms; (6) building up of theranostic platforms with specific responses to the tumor microenvironments; and (7) study of the long-term biosafety of Pdots prior to practical applications.

## Figures and Tables

**Table 1:** NIR-II Pdots for in vivo tumor fluorescence imaging and theranostics.

Pdots	*λ*_abs_ (nm)	*λ*_em_ (nm)	*λ*_ex_ (nm)	*Φ*_f_ (%)	ε/Weight (g L^−1^ cm^−1^)	Tumor Model	Application	Ref.
m-PBTQ4F	946	1123	808	3.2	n.a.	Mouse medulloblastoma tumor	FI	[54]
IR-Pttc	706	1008	793	4.9	n.a.	4T1 breast tumor	3D FI	[55]
IR-TPA	670	950	793	6.7	n.a.	4T1 breast tumor	3D FI	[55]
IR-TPE	702	1010	793	14	n.a.	4T1 breast tumor	3D FI	[55]
Pdots-C6	745	1055	808	0.6	n.a.	C6-glioma tumor	FI	[56]
Pdots-GnRH	710	1020	730	5.5	15.3	A2780-metastatic ovarian tumor	FI	[57]
L1057	980	1057	980	1.25	18	4T1 breast tumor	FI + PTT	[58]
TTQ-MnCO	808	1115	808	n.a.	n.a.	MCF-7 breast tumor	FI + PTT + Gas	[59]
PBQ45	1064	~1200	1064	0.048	27.5	Peritoneal carcinomatosis/4T1 tumor	FI/PTT	[60]
CSPN	700	893	835	1.76	n.a.	U373 glioma tumor	FI + PDT	[61]
